# TMT-based proteomic and bioinformatic analyses of human granulosa cells from obese and normal-weight female subjects

**DOI:** 10.1186/s12958-021-00760-x

**Published:** 2021-05-20

**Authors:** Chenchen Si, Nan Wang, Mingjie Wang, Yue Liu, Zhihong Niu, Zhide Ding

**Affiliations:** 1 Department of Histology, Embryology, Genetics and Developmental Biology, Shanghai Key Laboratory for Reproductive Medicine, School of Medicine, Shanghai Jiao Tong University, 200025 Shanghai, China; 2 Department of Gynecology and Obstetrics, Reproductive Medical Center, School of Medicine, Ruijin Hospital, Shanghai Jiao Tong University, 197 Ruijin 2nd Road, 200025 Shanghai, China

**Keywords:** Granulosa cells, Proteomic analysis, Obesity, Free fatty acids, Electron transport chain, Mitochondria

## Abstract

**Background:**

Increasing evidence supports a relationship between obesity and either infertility or subfertility in women. Most previous omics studies were focused on determining if the serum and follicular fluid expression profiles of subjects afflicted with both obesity-related infertility and polycystic ovary syndrome (PCOS) are different than those in normal healthy controls. As granulosa cells (GCs) are essential for oocyte development and fertility, we determined here if the protein expression profiles in the GCs from obese subjects are different than those in their normal-weight counterpart.

**Methods:**

GC samples were collected from obese female subjects (*n* = 14) and normal-weight female subjects (*n* = 12) who were infertile and underwent *in vitro* fertilization (IVF) treatment due to tubal pathology. A quantitative approach including tandem mass tag labeling and liquid chromatography tandem mass spectrometry (TMT) was employed to identify differentially expressed proteins. Gene Ontology (GO) and the Kyoto Encyclopedia of Genes and Genomes (KEGG) analyses were then conducted to interrogate the functions and pathways of identified proteins. Clinical, hormonal, and biochemical parameters were also analyzed in both groups.

**Results:**

A total of 228 differentially expressed proteins were noted, including 138 that were upregulated whereas 90 others were downregulated. Significant pathways and GO terms associated with protein expression changes were also identified, especially within the mitochondrial electron transport chain. The levels of free fatty acids in both the serum and follicular fluid of obese subjects were significantly higher than those in matched normal-weight subjects.

**Conclusions:**

In GCs obtained from obese subjects, their mitochondria were damaged and the endoplasmic reticulum stress response was accompanied by dysregulated hormonal synthesis whereas none of these changes occurred in normal-weight subjects. These alterations may be related to the high FFA and TG levels detected in human follicular fluid.

## Background

The prevalence of obesity is increasing all over the world, in consistence with the increase in obese subjects suffering from infertility [1]. Compared to age-matched normal-weight women, obese women have an increased risk of ovulatory subfertility and anovulatory infertility [2–4]. While ovarian stimulation has been used to overcome anovulation, obese subjects usually require higher doses of gonadotropins. Decreased oocyte retrieval, lower oocyte quality, and reduced rates of pre-implantation embryo development are generally observed in obese subjects, in addition to an increased risk of cycle cancellation or miscarriage compared with normal-weight subjects [5–9]. A considerable number of clinical reports have highlighted the negative effect of body mass index (BMI) on the success rate of fertility treatments. However, multiple studies reported no significant association between BMI and assisted reproductive outcomes [10–12]. This controversy indicates that the effect of obesity on oocyte quality and fertility outcome is somewhat complex, and underlying mechanisms remain to be elucidated [13].

Folliculogenesis requires a carefully orchestrated crosstalk between the oocyte and surrounding somatic cells [14]. Two granulosa cell types can be distinguished in the follicle, namely mural granulosa cells (GCs), lying on the basal membrane of the follicular wall, and cumulus GCs, which surround the oocyte [15]. These different GCs provide various molecules, such as sugars, amino acids, signaling factors, and nucleotides, to facilitate oocyte development [16], maturation, meiosis, and oocyte cytoskeleton alteration [17]. Hence, a better understanding of obesity-associated changes in GCs could allow us to identify the mechanisms underlying obesity-induced female infertility.

In the research field of obesity-related female infertility, omics studies were mostly limited to the analysis of follicular fluid or plasma in polycystic ovary syndrome (PCOS) [18, 19]. However, PCOS is a complex multigenic disorder, which is strongly influenced by various epigenetic and environmental factors [20]. It is therefore difficult to determine the effect of obesity on intrafollicular cell function. The aim of the current study was to investigate how protein expression changes in the GCs of obese women in which PCOS was ruled out as a possible confounder. The results strongly indicate that the GC proteomic expression patterns undergo significant alteration in the obese subjects.

## Methods

### Subject enrollment

This study enrolled subjects visiting the Reproductive Medical Center of Shanghai Ruijin Hospital *in vitro* fertilization (IVF) Clinics between September 2019 and January 2020. The study was approved by the local ethics committee of Shanghai Jiao Tong University School of Medicine. All subjects gave informed consent before participation. Twenty-six women with normal menstruation cycles were recruited. The Chinese-specific cut-offs for general adiposity were used, with normal weight as BMI 18.5-23.9 kg/m^2^ and general obesity as BMI values equal to or more than 28.0 kg/m^2^ [21]. Therefore, subjects with a normal BMI (20.38‒21.02 kg/m^2^) (*n* = 12) were assigned to the control group, and obese subjects with a BMI of 29.25–32.33 kg/m^2^ (*n* = 14) were assigned to the obesity group. The ages of the control group and the obesity group were 31.00 ± 1.86 years and 31.79 ± 1.37 years respectively. All participants were Chinese and experienced infertility due to tubal pathology. Subjects with endometriosis, PCOS, or other medical disorders that could affect folliculogenesis were excluded.

### Purification of GCs from follicular fluid

All subjects underwent controlled ovarian stimulation for IVF procedures using standard gonadotropin-releasing hormone antagonist protocols. After receiving human chorionic gonadotrophin injections for 34‒36 h, follicular fluid was obtained from follicles (16-20 cm in diameter) that contained granulosa cells (GCs). The purification procedure details are as follow: when the mature follicles were obtainable, the cumulus-oocyte complexes were separated from the follicles for IVF treatment, and the remaining follicular fluid was collected and centrifuged at 250×g for 10 min. Then the resulting pellet was resuspended with 1X PBS and subsequently layered on 40 % Percoll® (Sigma-Aldrich; Merck KGaA) followed by centrifugation at 450×g for 20 min at room temperature. After performing this density gradient centrifugation, the mixture was divided into four layers from the button to the top surface for separating red blood cells, Percoll, granulosa cells and follicular fluid with PBS. The granulosa cell layer was collected and washed with 1 × PBS at 250×g for 10 min. The pellet was resuspended with 1X PBS,and then the red blood cell lysate was added to a volumetric ratio of 1:3 and kept at 4℃ for 5 min. Finally, three wash steps were carried out using 1X PBS at 250×g for 1 min at room temperature, and purified GCs were collected.

### Total protein extraction

In order to establish protein content equivalence of 120 µg in each sample, the GCs of a single subject whose protein content was less than 120 µg were pooled with other GCs belonging to one or two subjects in the same group. This procedure generated 5 control and 5 obese samples in each group. Samples were ground individually in liquid nitrogen and lysed with lysis buffer, followed by ultrasonication on ice for 5 min. The mixture was then centrifuged at 12,000 × g for 15 min at 4 ℃, and the supernatant was collected. Extracts were reduced with 10 mM DTT for 1 h at 56 ℃ individually and were alkylated with iodoacetamide in the absence of light for 1 h at room temperature. The samples were then mixed with precooled acetone, followed by incubation and centrifugation. Next, the precipitate was collected, washed with cold acetone, and then the pellet was dissolved in dissolution buffer containing 0.1 M triethylammonium bicarbonate (TEAB, pH 8.5) and 6 M urea. Finally, the Bradford Protein Assay Kit (Beyotime Biotechnology, China) was used to determine the protein concentrations.

### Protein digestion and tandem mass tag (TMT) labeling

A total of 120 µg per protein sample was added to 100 µL of dissolution buffer. Thereafter, 1.5 µg trypsin and 500 µL of 100 mM TEAB buffer were added, followed by digestion at 37 °C for 4 h. Subsequently, 1.5 µg trypsin and CaCl_2_ were added, and the sample was digested overnight. Formic acid was mixed with the digested sample, and the pH was adjusted to < 3, and then the sample was centrifuged at 12,000 × *g* for 5 min. After the supernatant flowing through a C18 desalting column, the column was washed thrice (0.1 % formic acid, 4 % acetonitrile) and then eluted with elution buffer (0.1 % formic acid, 75 % acetonitrile). The eluents were collected and lyophilized. Next, 100 µL of 0.1 M TEAB buffer was added for reconstitution, followed by the addition of 41 µL of acetonitrile-dissolved TMT labeling reagent (ThermoFisher, Massachusetts, USA). After mixing the samples by shaking for 2 h, the reaction was stopped by the addition of 8 % ammonia. Then, labeling samples were desalted and lyophilized.

### Separation of fractions

To develop a gradient elution, mobile phase A (2 % acetonitrile, adjusted pH to 10.0) and B (98 % acetonitrile) were used. The lyophilized powder was dissolved in solution A. The sample was fractionated using a C18 column, and the column oven was set at 50 °C. At 214 nm, the eluates were monitored, followed by collection into a tube every minute, and eventually combination into 10 fractions. Under vacuum, all fractions were dried and then reconstituted in 0.1 % (v/v) formic acid in water.

### Liquid chromatography tandem mass spectrometry (MS) analysis

For transition library construction, shotgun proteomics analyses were conducted using an EASY-nLCTM 1200 UHPLC system (Thermo Fisher, Massachusetts, USA) coupled with a Q Exactive HF(X) mass spectrometer (Thermo Fisher, Massachusetts, USA). The Q Exactive HF(X) MS allowed operation in a data-dependent acquisition (DDA) mode.

### Identification and quantification of differentially abundant proteins

The resulting spectra from each run were searched against the SwissProt human database using the Proteome Discoverer v2.2 (ThermoFisher, Massachusetts, USA) search engine. A maximum of two miscleavage sites were allowed. Peptide spectrum matches (PSMs) with a credibility of more than 99 % were identified as PSMs. The identified proteins contained at least one unique peptide. PSMs and proteins with a false discovery rate (FDR) of no more than 1.0 % were retained. Differentially expressed proteins (DEPs) were identified using the limma package [22] with the following criteria: (1) fold change value > 1.2- or < 1/1.2, (2) *P* value < 0.05. DEP analysis was carried out in R 4.1.0.

### Bioinformatics

Gene Ontology (GO) annotation of identified proteins was performed using the UniProt-GOA database and InterProScan program version 5 [23]. Pathway annotation was carried out using the Kyoto Encyclopedia of Genes and Genomes (KEGG) database. Enrichment analysis of functional annotations was conducted using the R package clusterProfiler [24]. A *P* value < 0.05 was considered significant, and enriched GO terms or pathways were sorted by *P* values. Principle component analysis (PCA) and t-distributed stochastic neighbor embedding (t-SNE) clustering analyses [25] were conducted using the princomp() function or Rt-SNE package, respectively, in R. Hierarchical clustering analysis of DEPs was performed using the R gplots package. Protein-protein interactions (PPI) were derived using the STRING database [26] (https://string-db.org/). Network visualization of PPI was carried out using Cytoscape version 3.8.0 [27], and hub scores for each identified DEP were obtained using the cytohubba plugin [28]. The modules of hub genes were filtered using the MCODE plugin in Cytoscape with the following criteria: degree cut-off > 2, node score cut-off > 0.2, K-score > 2, and max depth = 10.

### Determination of lipids in serum and follicular fluid

Venous blood or follicular fluid samples were centrifuged at 3,000 *× g* for 10 min to isolate the upper layer for analysis. Serum sex hormones, including follicle-stimulating hormone, luteinizing hormone, estradiol, progesterone and testosterone, were analyzed using commercially available kits from the Unicel DXI 800 Access immunoassay system (Beckman Coulter). Total cholesterol, triglycerides, high-density lipoprotein, and low-density lipoprotein levels were determined using commercially available kits purchased from Randox Laboratories Ltd., Northern Ireland, United Kingdom. The assays were carried out on a Hitachi 7600–210 Automatic Biochemical Analyzer (Hitachi Corporation, Japan). Commercially available kits for free fatty acid (FFA) determination were purchased from Nanjing Jiancheng Bioengineering Institute (Jiangsu, China).

### Statistical analysis

All analyses were performed using R version 4.1.0. Normally distributed continuous data are presented as the mean ± SE and were compared using the Student’s *t*-test, while non-parametric data were presented as the median (interquartile range) and were compared using the Mann–Whitney U test, when appropriate. Correlations were evaluated using Spearman’s correlation coefficient. A two-sided *P*-value < 0.05 was considered statistically significant for all tests.

## Results

### Subject clinical characteristics

Descriptive statistics and ovarian stimulation characteristics were recorded (Table 1). There was no statistical difference in age, basal hormone levels, or ovarian stimulation procedure data between the two groups. The BMI in the obesity group ranged from 29.25 to 32.33 kg/m^2^, much higher than that in the control group (20.38‒21.02 kg/m^2^).

**Table 1:** Clinical parameters and ovarian stimulation in control and obese groups

	**Control group (*n*=12)**	**Obesity group (*n*=14)**	***P*‑value**
**Patients information**			
**Age (y)**	31.00±1.86	31.79±1.37	0.227
**BMI (kg/m2)**	20.75(20.38-21.02)	30.70(29.25-32.33)	<0.001
**Basal sex hormones (serum)**			
**FSH(IU/L)**	8.36±1.16	8.52±0.77	0.687
**LH(IU/L)**	3.87±1.21	4.25±1.14	0.423
**E2(pg/ml)**	40.50±9.06	34.93±8.40	0.117
**P(ng/ml)**	0.68±0.25	0.74±0.22	0.549
**TT (ng/ml)**	0.37±0.05	0.40±0.07	0.207
**Ovarian stimulation cycles**			
**Total FSH dose (IU)**	2806.25±368.52	2850.00±314.09	0.747
**Duration of stimulation (day)**	10.00(9.00-10.25)	10.00(9.00-10.75)	0.874
**E2 on trigger day (pg/ml)**	4096.00±1022.08	3987.29±1065.23	0.794
**LH on trigger day (IU/L)**	3.09±1.11	2.81±0.95	0.486
**P on trigger day(ng/ml)**	1.08±0.26	1.02±0.23	0.591

### Subject lipid parameters

Table 2 summarizes lipid levels in the serum and follicular fluid of all participants. FFA and triglycerides levels in follicular fluid and FFA levels in serum of obese women were significantly higher than those in matched normal-weight women. No differences were observed in total cholesterol, high-density lipoprotein, and low-density lipoprotein levels between the two groups.

**Table 2:** Lipids Levels of serum and follicular fluid in control and obese groups

	Control group(*n*=12)	Obesity group (*n*=14)	*P*‑value
**Serum**			
TC (mmol/L)	4.12±0.75	4.22±0.95	0.779
TG (mmol/L)	1.03±0.34	1.28±0.41	0.098
LDL (mmol/L)	2.48±0.48	2.41±0.49	0.715
HDL (mmol/L)	1.30±0.10	1.29±0.12	0.899
FFA (mmol/L)	0.37±0.12	0.47±0.12	0.042
**Follicular fluid**			
TC (mmol/L)	0.72±0.22	0.78±0.19	0.487
TG (mmol/L)	0.24(0.23-0.26)	0.29(0.26-0.32)	0.01
FFA (mmol/L)	0.32(0.31-0.34)	0.44(0.42-0.46)	<0.001

### Proteomics analysis

**Fig. 1:**
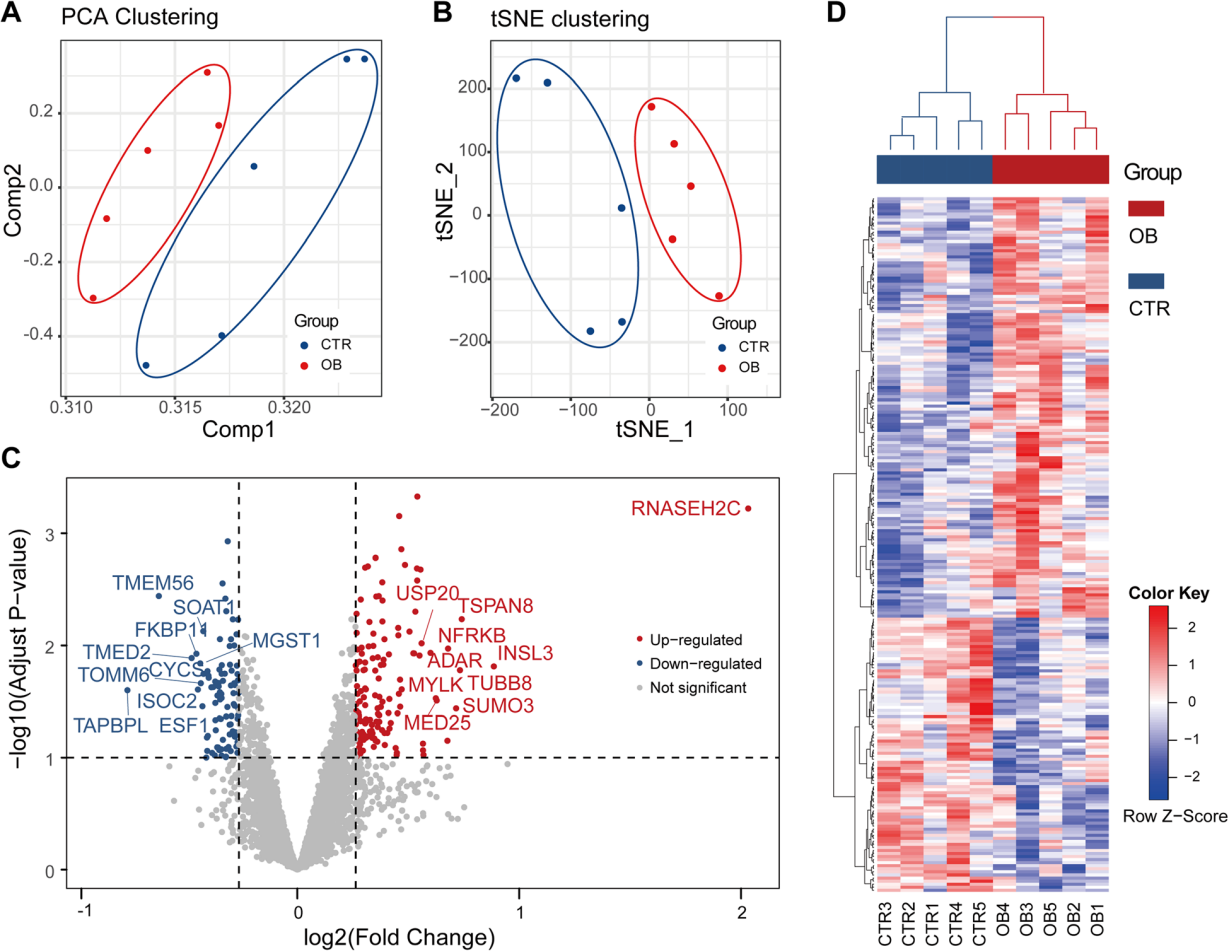
Quantitative analysis of proteomics data between control group and obese subjects. **a** Scatter plot showing the principle component analysis clustering of subjects. Each dot represents a subject and is colored according to subject groups indicated in the bottom-right corner of the plot. **b** Scatter plot showing the t-distributed stochastic neighbor embedding clustering of subjects. **c** Volcano plot of differentially expressed proteins (DEPs). The x-axis corresponds to the log2-transformed fold change of DEPs identified when comparing obese group (OB) versus control group (CTR). The y-axis corresponds to -log10-transformed *P* values. Upregulated and downregulated DEPs are colored in red and blue, respectively. The top 10 upregulated and downregulated DEPs are labeled. **d** Heatmap showing the hierarchical clustering of samples and DEPs. Samples of the OB and CTR groups are colored in red and blue, respectively

To obtain a global view of the GC proteome in normal-weight and obese subjects, sample clustering based on all identified proteins was performed. As shown in Fig. 1 a, PCA analysis revealed two significant clusters, which indicated distinct protein expression levels in obese subjects. The above result was further confirmed using t-SNE clustering analysis, which also showed two isolated clusters (Fig. 1b). Subsequently, DEPs between the two groups were screened. A total 228 DEPs were identified, including 138 upregulated and 90 downregulated proteins (Fig. 1 c). As shown in Fig. 1 c, the top 10 DEPs were highlighted and labeled, including RNASEH2C, INSL3, TAPBPL, TSPAN8, TUBB8, and SUMO3. Next, hierarchical clustering based on DEPs was performed, and the results revealed clusters similar to those of PCA and t-SNE analysis (Fig. 1d). The annotation and quantification information for the top 10 DEPs are presented in Table 3.

**Table 3:** List of differentially expressed proteins identified in GCs obtained from obese women and control group

**Protein Accession**	**Gene**	**Description**	**Fold change**	***P*-Value**
E9PN81	RNASEH2C	Ribonuclease H2 subunit C	4.09	<0.0001
P51460	INSL3	Insulin-like 3	1.85	0.02
Q9BX59	TAPBPL	Tapasin-related protein	0.59	0.03
P19075	TSPAN8	Tetraspanin-8	1.67	0.01
Q3ZCM7	TUBB8	Tubulin beta-8 chain	1.66	0.02
A8MU27	SUMO3	Small ubiquitin-related modifier 3	1.64	0.04
A0A024R3N2	NFRKB	Nuclear factor related to kappaB binding protein, isoform CRA_a	1.6	0.01
Q96GX5	MASTL	Serine/threonine-protein kinase greatwall	1.6	0.07
Q71SY5	MED25	Mediator of RNA polymerase II transcription subunit 25	1.55	0.03
Q15746	MYLK	Myosin light chain kinase, smooth muscle	1.54	0.03

### Changes in the mitochondrial electron transport chain in obese subject GCs

**Fig. 2:**
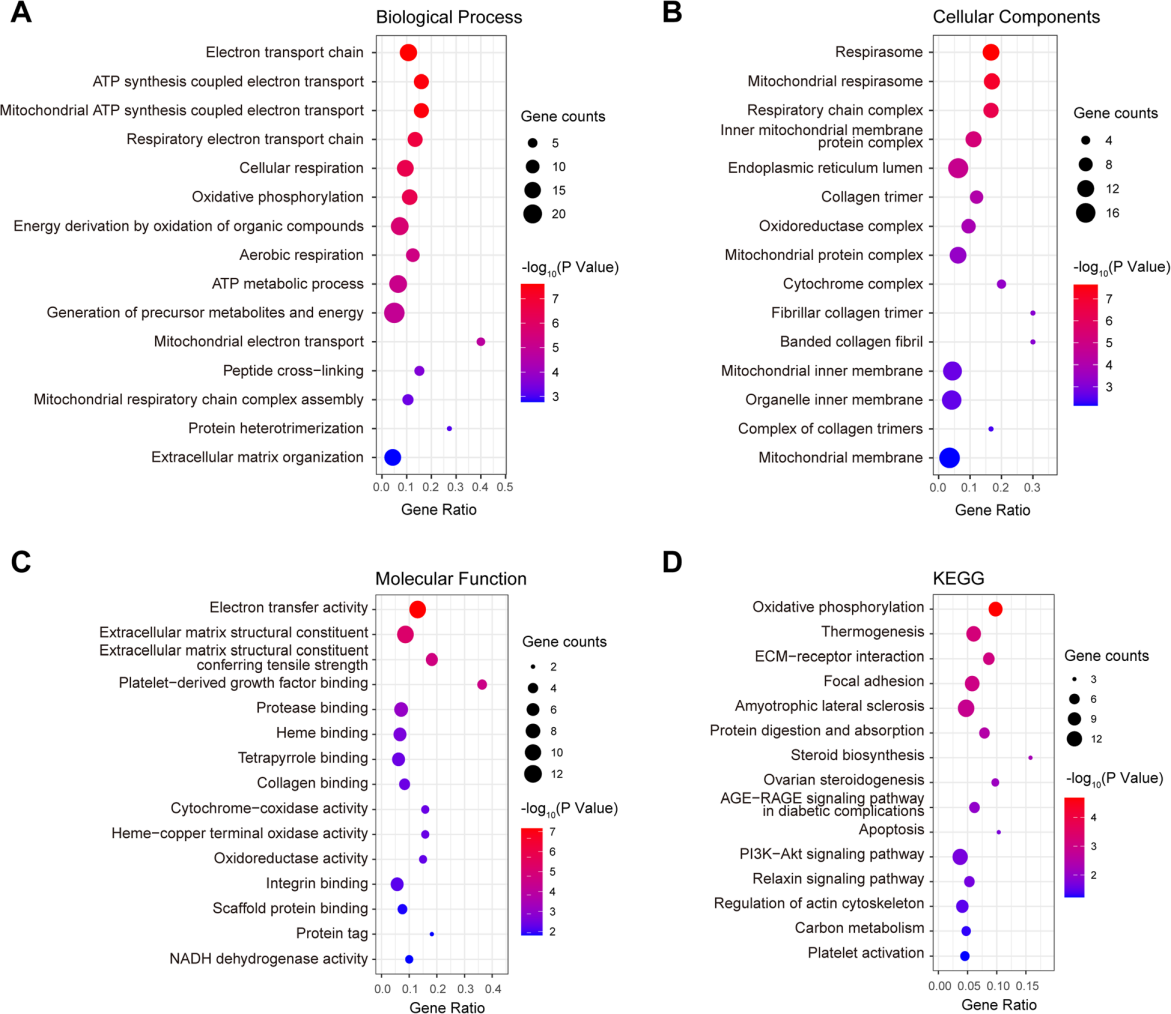
GO and KEGG enrichment of differentially expressed proteins (DEPs). Top 15 significantly enriched GO terms for (**a**) biological process, **b** cellular component, **c** molecular function, and (**d**) KEGG pathway analysis. Dot size represents DEP counts in corresponding GO term/pathway, and the dot color represents the -log10-transformed statistical *P* value. X axis corresponds to the gene ratio value. Gene Ontology (GO); the Kyoto Encyclopedia of Genes and Genomes (KEGG)

Functional enrichment analyses, including GO and KEGG, were carried out to investigate the roles of DEPs in GCs and to explore the effect of obesity on oocyte quality and fertility outcome. DEPs were enriched in biological processes related to the mitochondrial respiratory chain, including electron transport, ATP synthesis, cellular respiration, energy derivation by oxidation of organic compounds, and ATP metabolic process (Fig. 2 a). In the cellular component analysis, DEPs were found to be enriched in respirasome, mitochondrial respirasome, endoplasmic reticulum lumen, and other energy metabolism-related subcellular locations (Fig. 2b). Similar results were observed in molecular function analysis, in which electron transfer activity, protease binding, and oxidoreductase activity, among other terms, were significantly enriched (Fig. 2 c).

KEGG pathway analysis revealed that the most significantly enriched pathways included energy metabolism (oxidative phosphorylation, thermogenesis, and carbon metabolism), extracellular matrix (ECM) (ECM-receptor interaction, focal adhesion, and regulation of actin cytoskeleton), steroid hormone synthesis (steroid biosynthesis and ovarian steroidogenesis), diabetes complications (AGE-RAGE signaling pathway in diabetic complications), apoptosis, the PI3K-AKT signaling pathway, and the relaxin (INSL3) signaling pathway, among other terms (Fig. 2d).

### Key genes and modules based on the PPI network

**Fig. 3:**
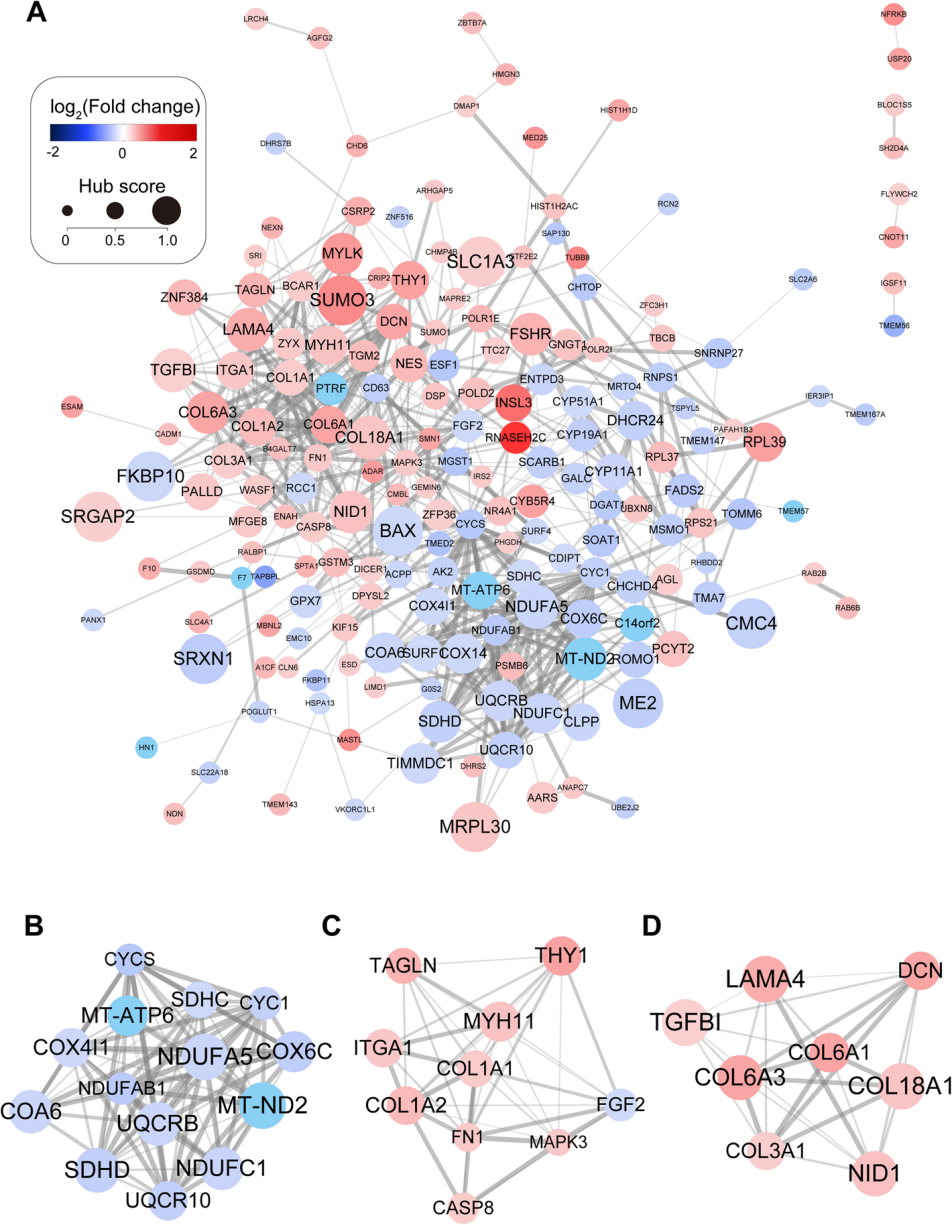
Protein-protein interactions (PPI) network of differentially expressed proteins (DEPs). **a** PPI network of all DEPs. DEPs are illustrated as dots, and edges between DEPs indicate potential interactions. Dot color corresponds to the log2-transformed fold change (obese group versus control group), while dot size and dot label size correspond to the clustering coefficient. Edge width corresponds to the combined interaction score predicted by the STRING database. **b**, **c**, **d** represent the three core modules identified by Cytoscape

We built the PPI network of DEPs using the STRING database. All DEPs were filtered into the DEP PPI network complex, which contained 189 nodes and 623 edges (Fig. 3 a). Among the 189 nodes, 11 central node genes were selected as hub genes with the criterium of filtering degree ≥ 20 (i.e., each node having more than 20 connections/interactions). The hub genes were *FN1, MAPK3, CYCS, CYC1, NDUFAB1, SDHC, COL1A1, COX4I1, FGF2, COL3A1*, and *UQCR10*. To identify the core gene modules that might play an important role in related pathways, core gene modules were also analyzed, and a total of three hub modules are shown in Fig. 3b-d. Functional enrichment analysis revealed that the genes in hub modules 1, 2, and 3 were mainly associated with “ATP synthesis,” “leukocyte migration”, and “extracellular matrix organization”, respectively (Fig. 3 a).

## Discussion

Obesity and infertility are inextricably linked, and their relationship has been increasingly recognized [29]. As a key factor, GCs play an important role in oocyte and follicle development. They secrete nutrients and hormones essential for primary and secondary follicle growth, providing a suitable microenvironment for oocyte meiosis and maturation [30, 31]. Therefore, a precise understanding of the effect of obesity on GCs may help to determine the mechanism of obesity-related female infertility or subfertility.

The exposure of ovarian cells to high levels of fatty acids could result in an inflammatory response within ovarian follicles [32], excessive ovarian androgen production through ineffective FFA β-oxidation in mitochondria [33], insulin resistance [34], and oxidative DNA damage caused by reactive oxygen species (ROS) reacting with DNA within the cell, including mitochondrial DNA (mtDNA) [35]. In the current study, the concentration of FFA and triglycerides in the follicular fluid of obese subjects was higher than that in control group. This was consistent with the results of our previous study, in which we found that FFA and triglycerides, as important biomedical indicators of abnormal lipid metabolism, were elevated in the serum and follicular fluid of obese subjects with PCOS and associated with lower embryo quality [36].

Mass spectrometry is widely used in proteomics for accurate and high-throughput analysis, allowing for the identification and quantification of proteins in complex samples with high sensitivity [37]. In this study, we used TMT-based high-throughput MS to investigate the proteome of GC samples from normal-weight and obese subjects. DEPs between the two groups were screened, and a total of 228 DEPs were identified, including 138 upregulated and 90 downregulated proteins. Significant pathways and GO terms associated with protein expression changes were also identified, especially those associated with the mitochondrial electron transport chain. With regard to the biological process category, almost all of the significant GO terms were associated with the mitochondrial respiratory chain.

Caloric excess and high-fat diet is the most common cause of obesity. High-fat diet can lead to nutritional excess, increased electron flux, rising oxidative stress, an accumulation of oxidized substrates, and eventually, mitochondrial injury [38]. Physiologically, the mitochondrial matrix flowing within the inner membrane, which hosts several copies mtDNA as well as components of the tricarboxylic acid pathway and electron transport chain, is the site of ATP production [39]. Excess FFA can increase mitochondrial activity, contributing to higher oxidative stress, which adversely affects the replication of mtDNA and leads to the dysfunction or dysregulation of key mtDNA gene products. Subsequently, mtDNA gene products control oxidative phosphorylation, thereby impairing the maturation and developmental potential of oocytes [40]. Damage to mitochondrial function caused by high concentrations of FFA has been confirmed by both *in vivo* and *in vitro* studies in multiple tissue and cell types [41–43]. In the current study, a significant correlation between high FFA in follicular fluid and mitochondrial dysfunction in GCs from obese subjects was observed. Among molecular function GO terms, electron transfer activity, which is essential for mitochondrial energy production, was listed as the most significantly perturbed function. In addition, cytochrome-c-oxidase activity, which is also key factor for the respiratory electron transport chain, was altered in GCs from obese subjects.

The ECM is a diverse mixture of molecules, influencing almost all aspects of the follicle development process, including migration, division, differentiation, cell death, and cell anchorage [44]. Various ECM types in the ovarian follicles have been characterized, including those associated with the follicular basal lamina, the stromal matrix of the thecal layers, the matrix of thecal blood vessels, and that of the cumulus oocyte complexes [45–47]. Changes relevant to the structure of the ECM were related to a large part of the obtained molecular function GO terms, including changes in extracellular matrix structural constituent, extracellular matrix structural constituent conferring tensile strength, protease binding, collagen binding, and scaffold protein binding. The current study findings indicated that obesity and high FFA levels may affect the function of ovarian GCs via alteration of the ECM.

The regulation of endoplasmic reticulum homeostasis is indispensable during folliculogenesis and oocyte maturation [48]. Some physiological and pathological conditions, such as glucose deprivation, inflammation, oxidative stress, elevated FFA levels, aberrant Ca^2+^ regulation, and hypoxia disrupt endoplasmic reticulum function, which results in the accumulation of unfolded or misfolded proteins within the endoplasmic reticulum, leading to the induction of endoplasmic reticulum stress [49]. Fatty acid-induced endoplasmic reticulum stress in mouse cumulus-oocyte complexes impairs protein secretion and mitochondrial activity, resulting in abnormal embryo development [50]. Another mouse study also demonstrated a relationship between obesity and endoplasmic reticulum stress in cumulus-oocyte complexes, which was associated with reduced mitochondrial membrane potential, high autophagy levels, and high intracellular lipid levels [51]. The mechanisms underlying endoplasmic reticulum changes in the GCs of obese female subjects in this study may be similar to those reported in animal studies [50, 51].

Regarding the cellular component category, the major enriched terms were related to the mitochondria, ECM, and endoplasmic reticulum, which were also consistent with the results for the biological process and molecular function categories. The mitochondria-related terms included mitochondrial respirasome, respiratory chain complex, cytochrome complex, and oxidoreductase complex. ECM terms included collagen trimer, fibrillar collagen trimer, banded collagen fibril, and complex of collagen trimers. Endoplasmic reticulum terms included endoplasmic reticulum lumen.

Several studies have shown that oxidative stress caused by increased levels of ROS can induce GC apoptosis, resulting in decreased estradiol 17β levels, lower ovulation rates, and compromised oocyte quality [52]. In the KEGG pathway analysis, energy metabolism and ECM-related pathways were among the most significantly enriched, which were consistent with GO results. Thus, oxidative stress caused by mitochondrial dysfunction and a high fatty acid environment may affect steroid synthesis, in turn affecting the quality of oocytes.

In patients with PCOS, advanced glycation end-product (AGE) levels were significantly increased compared with those in matched normal-weight controls [53, 54]. AGEs can alter insulin receptor substrate and AKT phosphorylation, thereby stimulating the downstream PI3K signaling cascade and compromising the effects of insulin signaling [55, 56]. Excessive deposition of AGEs can impair the ECM structure, altering its biochemical characteristics and associated metabolism [57]. The binding of AGE to its receptor RAGE produces NAPDH, which enhances oxidative stress, activates the NF-κB signaling pathway and further stimulates the production of cytokines and other factors. It finally contributes to cellular damage and even apoptosis [58, 59]. Moreover, the PI3K-AKT signaling pathway was associated with human GC damage and apoptosis [60, 61]. An interesting result of the KEGG pathway analysis was the significant enrichment of the AGE-RAGE signaling pathway in the current study. This is the first to demonstrate the association between AGEs and impaired GC function in obese non-PCOS subjects. It suggests that obesity-related infertility or subfertility is closely associated with the metabolic, inflammatory, structural, and functional changes in GCs.

TSPAN8 is a cell surface glycoprotein, which forms complexes with integrins. Previous genome-wide association studies identified TSPAN8 loci, which was correlated with an increased risk of type 2 diabetes (T2D) in humans [62–64]. Close examination of the haploblock structure with the inclusion of additional SNPs suggested that TSPAN8 is likely to be a T2D causal gene [65]. Genetic ablation of TSPAN8 resulted in a reduction of body weight in mice fed a normal diet as well as in a resistance to body weight gain upon high-fat and high-carbohydrate diet feeding [65]. Small ubiquitin-like modifier 3 (SUMO3) is a member of the SUMO family of eukaryotic proteins. Subcutaneous adipose tissue from cows incubated with insulin exhibited an upregulation of SUMO3, in parallel to an increased expression of other key genes associated with insulin signaling, adipogenesis, and lipogenesis [66]. In the field of reproduction, previous literature reported that excessive SUMOylation (SUMO1/2/3) was a marker of defective spermatozoa [67]. INSL3 is a member of the insulin-like hormone superfamily and is mainly produced in gonadal tissues. Within the follicle, INSL3 acts via its G protein-coupled receptor, RXFP2, in an autocrine/paracrine manner to drive the production of the major steroid precursor androstenedione as well as its conversion into estrogen by GCs [68]. MED25 was previously shown to play a critical role in the endoplasmic reticulum stress response [69] Here, a number of the DEPs identified in the current work, including TSPAN8, SUMO3, INSL3, and MED25, were implicated in obesity-related infertility or subfertility. In addition, INSL3 was one of the most differentially expressed proteins, which was consistent with the enrichment of the relaxin signaling pathway in KEGG analysis. Other major DEPs, including RNASEH2C, TAPBPL, TUBB8, NFRKB, MASTL, and MYLK, have never been reported to be associated with infertility, metabolic disorders, or hormone imbalances. Thus, their role in the occurrence and development of female infertility requires further attention.

## Conclusions

In summary, TMT-based proteomic and bioinformatic analyses indicated that mitochondrial damage, the endoplasmic reticulum stress response as well as dysregulated hormonal synthesis were exhibited in GCs obtained from obese subjects whereas none of these changes occurred in normal-weight subjects. These alterations may be related to the high FFA and TG levels detected in human follicular fluid. Therefore, the current research provides valuable insight for the further etiological investigation of obesity-related infertility or subfertility and the identification of therapeutic targets.

## Data Availability

The datasets used and analyzed during the current study are available from the corresponding author on reasonable request.

## Abbreviations

BMI: Body mass index
TC: Total cholesterol
TG: Triglycerides
HDL: High-density lipoprotein
LDL: Low-density lipoprotein
FFA: Free fatty acid
PCA: Principle component analysis
t-SNE: t-distributed stochastic neighbor embedding
PPI: Protein-protein interactions
PSMs: Peptide spectrum matches
GCs: Granulosa cells
CTR: Control group
OB: Obesity group
IVF: In vitro fertilization
TEAB: Triethylammonium bicarbonate
DEP: Differentially expressed protein
GO: Gene Ontology
KEGG: Kyoto Encyclopedia of Genes and Genomes
SY: Symmetry
PCOS: Polycystic ovary syndrome
mtDNA: Mitochondrial DNA
ROS: Reactive oxygen species
ECM: Extracellular matrix
ER: Endoplasmic reticulum
SUMO: Small ubiquitin-related modifier
AGE: Advanced glycation end-product
MF: Molecular function
CC: Cellular component
BP: Biological process
MS: Mass spectrometry
TMT: Tandem mass tag
OS: Oxidative stress
